# The Co-occurrence of Multiple Parathyroid Adenomas: A Case Report

**DOI:** 10.7759/cureus.30854

**Published:** 2022-10-29

**Authors:** Rusul Alani, Andeh Awai, Divya Yetukuri, Vijaykumar Patel

**Affiliations:** 1 Medical School, Ross University School of Medicine, St. Michael, BRB; 2 General Surgery, Wellstar Atlanta Medical Center, Atlanta, USA

**Keywords:** intraoperative pth monitoring, multiple parathyroid adenomas, primary hyperparathyroidism, parathyroid adenoma, parathyroidectomy, parathyroid

## Abstract

Solitary parathyroid adenoma is the most common cause of primary hyperparathyroidism; however, multiple parathyroid adenomas are an uncommon phenomenon. In this case report, we discuss a patient presenting with two concurrent ipsilateral parathyroid adenomas, and we review the literature. A 61-year-old African American female with a history of hypertension and kidney stones presented for the evaluation of hypercalcemia. Elevated serum calcium of 11 mg/dL was found on routine laboratory tests and low25 hydroxyvitamin-D level. Parathyroid hormone (PTH) was elevated at 172.5 pg/mL and increased to 443 pg/mL after correction of vitamin D deficiency. Renal function tests and thyroid function tests revealed normal findings. Imaging studies with 99mTc-Sestamibi scintigraphy raised concern for the possibility of either a parathyroid adenoma or hyperplasia. Minimally invasive parathyroidectomy with intraoperative PTH monitoring was planned. During surgery, the right superior parathyroid gland was found to be enlarged and was excised. However, intraoperative PTH monitoring showed an initial decrease to 203 pg/mL from a baseline of 443 pg/mL and a subsequent increase to 293 pg/mL suggesting the persistence of hyperparathyroidism. Further exploration of the neck revealed an enlarged right inferior parathyroid gland and two normal left parathyroid glands. Intraoperative PTH monitoring then revealed an appropriate decrease in PTH level to 56 pg/mL 10 minutes after excision of the right inferior parathyroid gland. More than a 50% decrease in PTH was achieved, and further exploration of the opposite side revealed no evidence of four-gland hyperplasia. Pathology reported two concurrent right superior and inferior parathyroid adenomas. Successful and curative parathyroidectomy for primary hyperparathyroidism was achieved. Although rare, multiple parathyroid adenomas occur in a significant minority of cases. Intraoperative PTH monitoring along with preoperative imaging provides guidance for curative parathyroidectomy. Additionally, more sensitive imaging such as four-dimensional computed tomography scans could lead to better localization, visualization, and identification of the second parathyroid adenoma.

## Introduction

Solitary parathyroid adenoma is the most common cause of primary hyperparathyroidism; accounting for 85-90% of all cases [1]. However, multiple parathyroid adenomas are reported to occur in 2-11% of all primary hyperparathyroidism cases making it a rare entity [2]. Other causes of primary hyperparathyroidism include parathyroid hyperplasia (15% prevalence) and parathyroid carcinoma (1% prevalence) [1-3]. Primary hyperparathyroidism is defined as abnormally high serum parathyroid hormone (PTH) levels due to overactivity of the parathyroid gland. Typically, humans have four parathyroid glands, which are posterior to the thyroid gland, and they each normally measure 5x3x1 mm and weigh less than 50 mg [3]. In patients with parathyroid adenoma, the affected parathyroid gland is enlarged as it is hyperactive, and parathyroid adenoma is considered a benign neoplasm. Moreover, elevated PTH levels will then cause serum calcium levels to rise as PTH promotes bone resorption, reabsorption of calcium in the kidney, and absorption of calcium in the small intestine.

It is important to note that a large cohort of parathyroid adenoma patients can present asymptomatically or with nonspecific symptoms such as fatigue, depression, irritability, anorexia, constipation, kidney stones, or confusion due to hypercalcemia. Thus, hypercalcemia on routine laboratory tests should be further evaluated with a PTH level to confirm the diagnosis of primary hyperparathyroidism. Imaging studies can then be used to help determine the cause of the primary hyperparathyroidism being either parathyroid adenoma, hyperplasia, or carcinoma. Neck ultrasound and Sestamibi neck scan are considered first-line investigations of the parathyroid gland, and these imaging studies are more sensitive than other modalities in localizing and visualizing the parathyroid gland. However, these current imaging tests do not provide a definitive method to differentiate the cause of the primary hyperparathyroidism. Additional testing with an abdominal computed tomography scan or ultrasound can be considered to check for calcium deposits and kidney stones as complications of primary hyperparathyroidism. To rule out secondary causes of hyperparathyroidism, 25 hydroxyvitamin-D levels should be tested as vitamin D deficiency causes hypocalcemia, which then stimulates the parathyroid glands to produce more PTH. Chronic kidney disease is also associated with hyperparathyroidism and biochemical abnormalities, so renal function tests should be included in these patients. to support the diagnosis of primary hyperparathyroidism, 24-hour urine calcium levels and bone densitometry to measure bone loss, as hyperparathyroidism can cause osteoporosis, can be used. Yet, these tests will only confirm the presence of hyperparathyroidism and hypercalcemia but not the presence of parathyroid adenoma, hyperplasia, or carcinoma. Moreover, thyroid adenoma also presents as solitary nodules, and it should be differentiated from parathyroid adenoma [4]. Definitive treatment of primary hyperparathyroidism is with surgical resection of the affected parathyroid gland and parathyroidectomy. However, medical therapy with cinacalcet, which reduces both PTH and calcium, can be used in poor surgical candidates. If left untreated, parathyroid adenoma can cause cardiac arrhythmia, parathyroid crisis, and even death due to hypercalcemia [5]. 

In this case report, we present a case of primary hyperparathyroidism due to two parathyroid adenomas in which the patient underwent minimally invasive parathyroidectomy with intraoperative PTH monitoring. The role of intraoperative PTH monitoring is to ensure that the parathyroid adenoma is removed and that a potential second adenoma or four-gland hyperplasia is not missed. The half-life of PTH is three to five minutes, and to indicate surgical success, a 50% decrease in PTH level is expected after 10 minutes of resection of the affected parathyroid gland. A successful and curative parathyroidectomy results in eucalcemia at six months postoperatively and, in most patients, lifelong eucalcemia is expected [6]. 

## Case presentation

A 61-year-old African American female with a history of hypertension and kidney stones presented for the evaluation of hypercalcemia. The patient was noted to have hypercalcemia on routine laboratory tests by her primary care physician. Past medical history was significant for chronic hypertension, nephrolithiasis, deep venous thrombosis that was previously treated with apixaban anticoagulant therapy, chronic back pain, and uterine fibroids. The patient reported smoking cessation five years ago, and her smoking use included tobacco and other illicit drugs. The patient also reported occasional alcohol use. Family history was negative for hypercalcemia, familial isolated hyperparathyroidism, and multiple endocrine neoplasia syndromes. The patient reported no allergies. Medications included albuterol 90 mcg/actuation inhaler, cyclobenzaprine 10 mg, ibuprofen 800 mg, and lisinopril 5 mg. The patient denied headache, fatigue, abdominal pain, excessive urination, bone pain, joint pain, depression, and memory loss. Vital signs were as follows: blood pressure: 126/84 mmHg, heart rate: 88 pulses/min, temperature: 97.7 °F, respiratory rate: 16 breaths/min, and oxygen saturation: 97%. On physical examination, the Head, Eyes, Ears, Nose, and Throat (HEENT) exam showed no abnormalities. The neck was without any swellings or masses, and the trachea was in the central position. On chest examination, chest expansion was symmetrical, and clear breath sounds were heard on auscultation of all lung fields with normal respiratory effort. On cardiovascular examination, regular rate and rhythm were appreciated with normal S1 and S2 heart sounds; no murmur, rub, or gallop was noted. All the peripheral arterial pulses were palpable with strong and regular pulses. The abdomen was soft and nontender to palpation; bowel sounds were normoactive. On examination of the extremities, no pedal edema was identified. No pallor or skin lesions were noted on integumentary system examination. On neurological examination, the patient was alert and oriented to person, place, and time. A cranial nerve examination revealed normal findings, and no motor or sensory deficits were detected.

**Figure 1:**
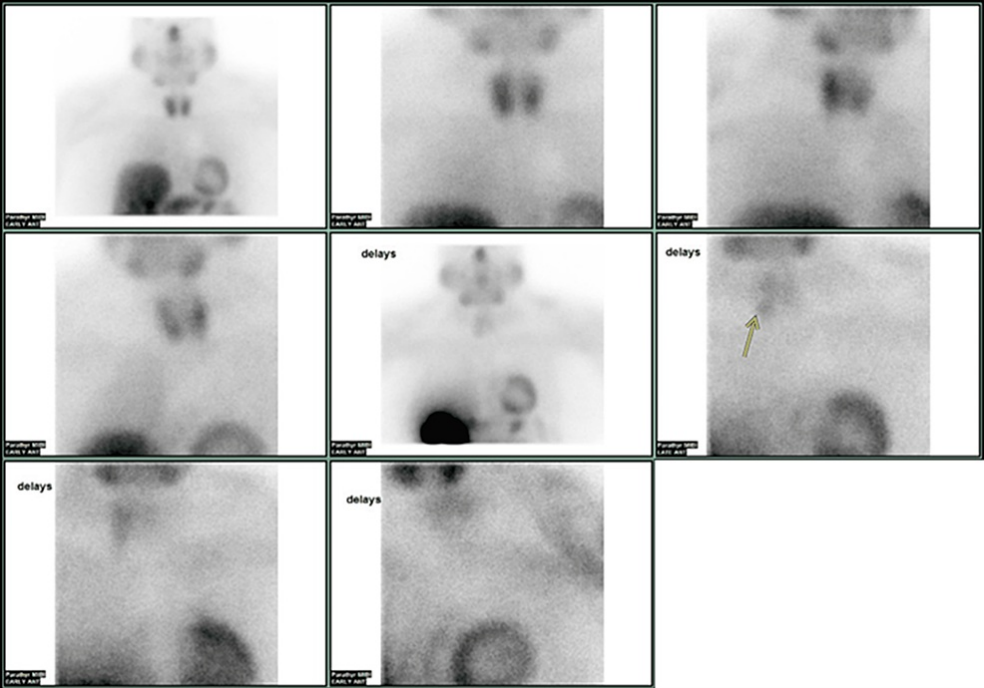
99mTc–Sestamibi Scintigraphy Sestamibi scan showing abnormal parathyroid findings concerning for either a parathyroid adenoma or hyperplasia in the region of the mid and inferior right lobe of the thyroid gland.

**Figure 2:**
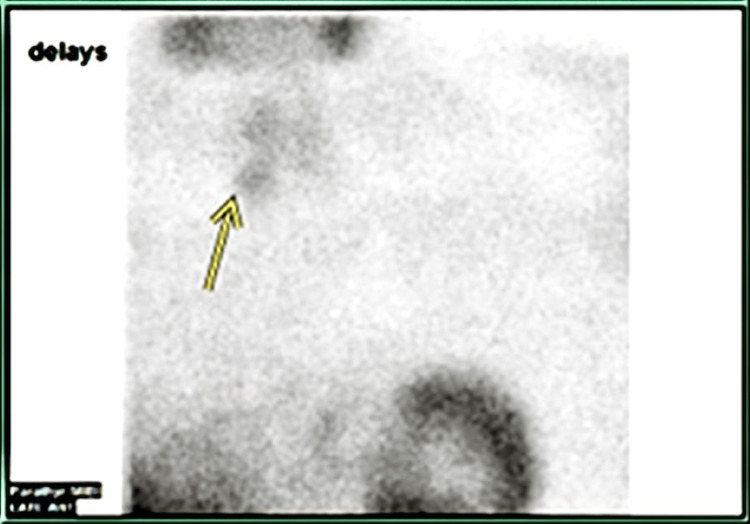
99mTc–Sestamibi Scintigraphy Sestamibi scan showing abnormal parathyroid findings concerning for either a parathyroid adenoma or hyperplasia in the region of the mid and inferior right lobe of the thyroid gland. (Image derived from Figure 1 for better visualization)

The patient was evaluated by an endocrinologist and was noted to have elevated serum total calcium of 11 mg/dL (normal range: 8.9-10.3 mg/dL) with an elevated PTH of 172.5 pg/mL (normal range: 12-88 pg/mL). 25 hydroxyvitamin-D level was low at 22 ng/mL (normal range: 30-100 ng/mL), and it was corrected with cholecalciferol. However, PTH was still elevated and increased to 443 pg/mL after the correction of vitamin D deficiency. Twenty-four-hour urine calcium level was elevated at 352 mg/24 h (normal range: 100-300 mg/24 h). Ionized calcium was elevated at 1.5 mmol/L (normal range: 1.12-1.32 mmol/L). Blood urea nitrogen, creatinine, glomerular filtration rate, and thyroid stimulating hormone were normal. The detailed laboratory results are reported in Table 1. Furthermore, the patient underwent 99mTc-Sestamibi scintigraphy, and the results were concerning for either a parathyroid adenoma or hyperplasia in the region of the mid and inferior right lobe of the thyroid gland (Figures 1, 2). The patient was planned to undergo minimally invasive parathyroidectomy with intraoperative PTH monitoring. 

**Table 1:** Complete Work-up and Laboratory Results MCV: mean corpuscular volume; MCH: mean corpuscular hemoglobin; MCHC: mean corpuscular hemoglobin concentration; RDW: red cell distribution width; ALT: alanine aminotransferase; SGPT: serum glutamic-pyruvic transaminase; AST: aspartate aminotransferase; SGOT: serum glutamic-oxaloacetic transaminase; GFR: glomerular filtration rate; TSH: thyroid stimulating hormone; Cpk: creatine phosphokinase

	Reference Range and Units	Value
Complete Blood Count		
WBC Count-Blood	3.8-10.7 K/mcL	8.6
RBC Count-Blood	3.73-4.96 M/mcL	4.42
Hemoglobin-Blood	11.3-15.0 g/dL	13.1
Hematocrit-Blood	33.6-44.6%	40.8
MCV	81-100.2 fL	92.0
MCH	26.1-34.5 pg	29.7
MCHC	31.5-35.0 g/dL	32.2
RDW	11.5-16.1%	13.8
Platelet Count-Blood	148-362 K/mcL	182
Platelet MPV	6.7-10.5 fL	9.4
Biochemical Tests		
Sodium-Serum	132-144 meq/L	135
Potassium-Serum	3.4-5.1 meq/L	4.6
Chloride-Serum	101-111 meq/L	103
Co2 Content-Serum	22-32 meq/L	25
Anion Gap	1-13	7
Glucose,Casual-Serum	70-125 mg/dL	95
Urea Nitrogen-Serum	8-22 mg/dL	19
Creatinine-Serum	0.4-1.0 mg/dL	0.8
Osmo,Calculated	275-300 mOsm/kg	272
Calcium,Total-Serum	8.9-10.3 mg/dL	11.0
Calcium,Albumin Adjusted	8.9-10.3 mg/dL	11.0
Protein,Total-Serum	6.0-8.3 g/dL	6.2
Albumin,Bcg-Serum	3.5-5.0 g/dL	3.5
ALT (SGPT)	14-54 U/L	26
AST (SGOT)	10-42 U/L	30
Bilirubin,Total-Serum	0.3-1.6 mg/dL	0.4
Bilirubin,Direct-Serum	0.1-0.4 mg/dL	0.1
Alkaline Phosphatase	38-141 U/L	83
GFR Calculated	>60 mL/min/1.73 mE2	>60
Vitamin D, 25-Hydroxy,Total	30-100 ng/mL	22
Parathyroid Hormone,Intact	12.0-88.0 pg/mL	172.5
TSH	0.34-5.60 ulU/mL	0.47
Cpk,Total-Serum	38-234 U/L	53
Calcium, Total-Urine	mg/dL	32
Calcium-24Hr-Urine	100-300 mg/24hrs	352
Ionized Calcium	1.12-1.32 mmol/L	1.50

**Figure 3:**
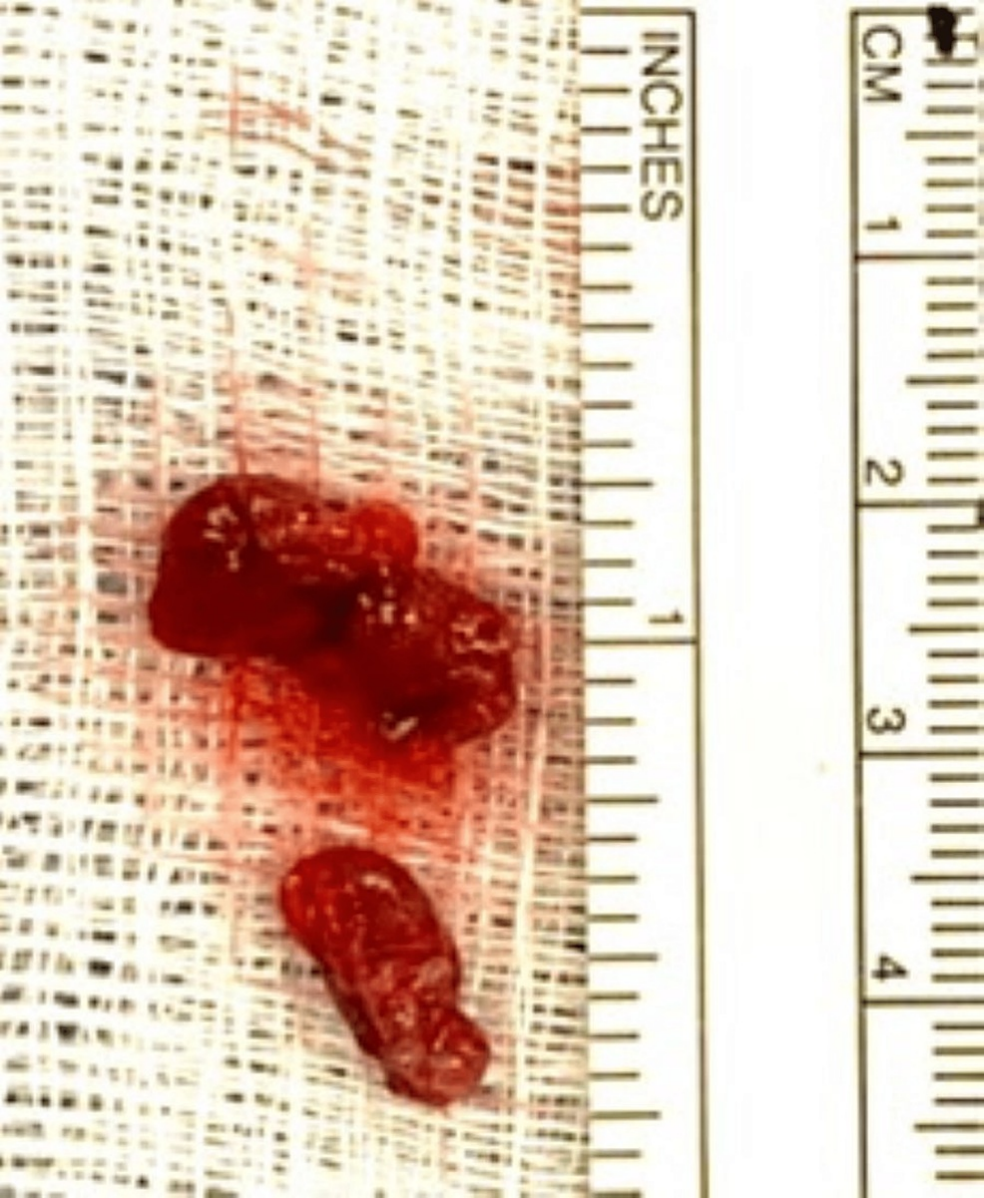
Right Superior Parathyroid Gland Excised right superior parathyroid gland measuring about 15x13 mm and weighing 600 mg.

**Figure 4:**
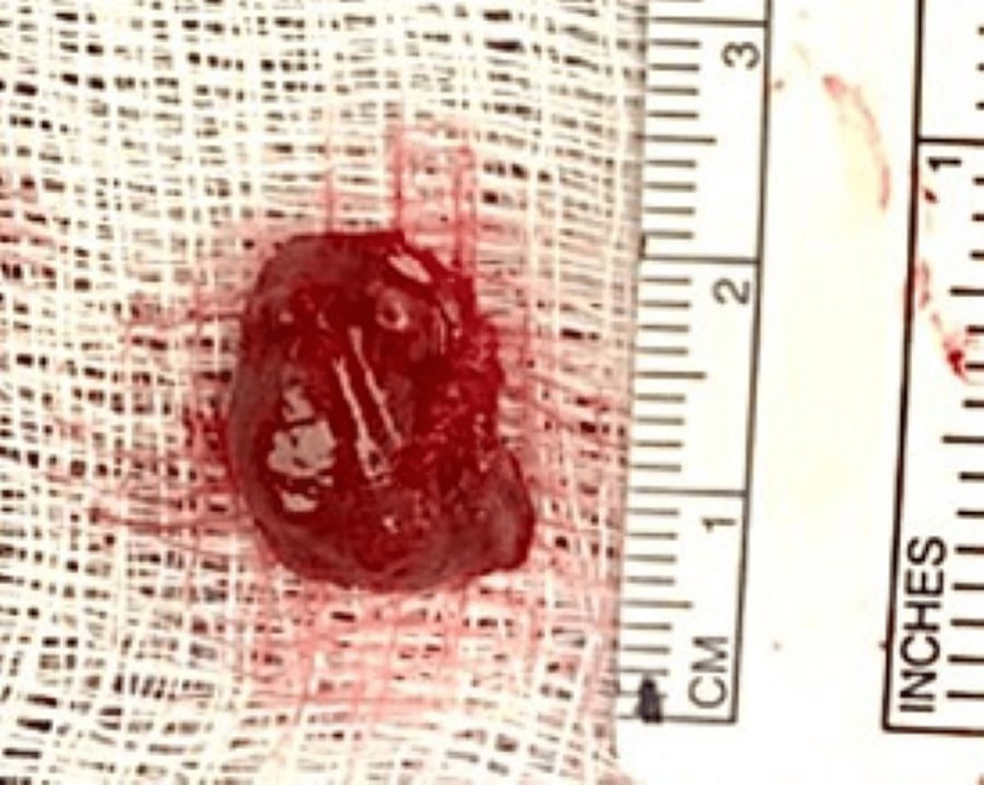
Right Inferior Parathyroid Gland Excised right inferior parathyroid gland measuring 14x12x6 mm and weighing 800 mg.

Intraoperatively, exploration of the right side of the neck revealed an enlarged superior parathyroid gland at the level of the thyroid isthmus. The right superior parathyroid gland was excised and sent for pathological evaluation (Figure 3). The PTH level after the removal of the right superior parathyroid gland dropped to 203 pg/mL from a baseline of 443 pg/mL, then after five minutes, PTH increased to 269 pg/mL and then slightly decreased to 253 pg/mL after 10 minutes. The pathologist noted hypercellular parathyroid gland consistent with parathyroid adenoma measuring about 15x13 mm and weighing 600 mg. PTH was evaluated again after 60 minutes, and it was 293 pg/mL suggesting persistent hyperparathyroidism. Further exploration of the right and left side of the neck revealed an enlarged right inferior parathyroid gland and two normal left parathyroid glands. The enlarged right inferior parathyroid gland was excised and sent to pathology (Figure 4). PTH level after five minutes dropped to 104 pg/mL and to 56 pg/mL after 10 minutes. The pathologist noted hypercellular parathyroid gland consistent with parathyroid adenoma measuring 14x12x6 mm and weighing 800 mg. 

The final pathology report revealed that both specimens were similar and represented enlarged hypercellular parathyroid glands with little stromal fat and increased intracytoplasmic fat. No atypical features were noted. Therefore, these microscopic features were consistent with two parathyroid adenomas. 

## Discussion

Parathyroid adenomas appear as a single adenoma in the majority of the patients with primary hyperparathyroidism. Laboratory values of elevated PTH levels due to a single adenoma occur in 85-90% of cases versus 15-20% resulting from multiple adenomas or hyperplasia [1]. In our study, we present a case with ipsilateral adenomas in the right superior and inferior parathyroid glands. The pathogenesis of multiple parathyroid adenomas is not well understood, but in our study, we notice that there is an equal contribution of the elevation of PTH from both adenomas. 

The etiology of parathyroid adenomas has yet to be eluded but a common genetic mutation, cyclin D1/PRAD1 gene, leading to the overproduction of PTH was identified [5]. The parathyroid glands are derived from the endoderm of the third and fourth pharyngeal pouches. The fourth pharyngeal pouch forms the superior parathyroid glands, and the third pharyngeal pouch forms the inferior parathyroid glands. During embryological formation, the parathyroid glands separate from the pharyngeal wall and descent to the neck, and attach to the posterior wall of the thyroid gland. In our case during the parathyroidectomy, we had an incidental finding of a rigid thyroid gland. Due to the long descent pathway, ectopic parathyroid tissue can be found anywhere along its descent [7-8]. The possibility of ectopic tissue being present in the thyroid can come into question. In this case, a biopsy of the rigid thyroid can be considered to confirm ectopic parathyroid tissue. If results confirm ectopic parathyroid tissue, then a thyroidectomy can be considered.

A normal parathyroid gland typically weighs less than 50 mg [3]. In our case, the two parathyroid adenomas weighed 600 mg and 800 mg. Also, the two adenomas had an independent effect on the PTH levels. When the first adenoma in the right superior parathyroid gland was excised, the PTH levels went from 443 pg/mL to 203 pg/mL. There was a 50% reduction, but repeat PTH levels began to increase instead of showing a significant decline. Once the second parathyroid gland, the right inferior parathyroid gland, was removed, PTH decreased from 293 pg/mL to 56 pg/mL indicating a successful parathyroidectomy. 

Parathyroidectomy is the treatment of choice for parathyroid adenomas. Due to the critical vasculature and nerves in proximity to the parathyroid glands, parathyroidectomy can result in recurrent laryngeal nerve injury or bleeding from the internal carotid or internal jugular vein. The incidence of hematomas is 0.5%. Additionally, when closing the platysma muscle and the strap muscles, they can adhere to the larynx leading to difficulty swallowing, but the incidence is very low. The vagus nerve is also at risk of injury during carotid sheath exploration. The inferior thyroid artery supplies the parathyroid glands, and the recurrent laryngeal nerve is in close proximity to the inferior thyroid artery where the recurrent laryngeal nerve may be injured. The recurrent laryngeal nerve has a higher probability of being affected in the resection of superior parathyroid adenomas since these tend to lean posteriorly in proximity to the inferior thyroid artery [9]. 

Furthermore, as seen in our patient, imaging with a Sestamibi neck scan was not able to definitively identify the secondary adenoma or the cause of the primary hyperparathyroidism being adenoma or hyperplasia. While intraoperative PTH is a great confirmatory tool for identifying any primary adenoma and remaining secondary adenomas or hyperplastic parathyroid glands in real-time, surgeons are unable to plan ahead. More sensitive imaging could lead to shorter operating time and potentially avoid the need for bilateral neck exploration. In a study from the Department of Surgery, Massachusetts General Hospital, -dimensional computed tomography scans were shown to be the most sensitive diagnostic tool in lateralizing the possible adenoma [10]. This test is highly accurate in localizing the parathyroid glands, and it is especially useful in localizing glands that are residing in uncommon areas such as behind the great vessels, behind the esophagus, or in the chest. 

## Conclusions

Although rare, multiple parathyroid adenomas occur in a significant minority of cases. Intraoperative PTH monitoring along with preoperative imaging provides guidance for a curative parathyroidectomy. As seen in this patient, imaging was not able to definitively identify the second adenoma or the cause of the primary hyperparathyroidism being either an adenoma or hyperplasia. More sensitive imaging such as four-dimensional computed tomography scans could lead to more accurate identification of solitary versus multiple parathyroid adenomas and thus potentially shorten the operating time and possibly avoid the need for bilateral neck exploration. Further studies are needed to evaluate the role of a four-dimentional computed tomography scan, which is not currently widely available at most institutions.

## References

[1] Tzikos G, Polyzonis M, Milias K (2021). Double ipsilateral parathyroid adenomas, with one supernumerary and ectopic at the same time: a case report. J Med Case Rep.

[2] Ilyicheva EA, Bersenev GA (2020). Surgical treatment of double parathyroid adenomas in primary hyperparathyroidism: a clinical case. Int J Surg Case Rep.

[3] Guilmette J, Sadow PM (2019). Parathyroid pathology. Surg Pathol Clin.

[4] Mulita F, Anjum F (2022). Thyroid adenoma. StatPearls [Internet].

[5] Wolfe SA, Sharma S (2022). Parathyroid adenoma. StatPearls [Internet].

[6] Naik AH, Wani MA, Wani KA, Laway BA, Malik AA, Shah ZA (2018). Intraoperative parathyroid hormone monitoring in guiding adequate parathyroidectomy. Indian J Endocrinol Metab.

[7] Kroeker TR, Stancoven KM, Preskitt JT (2011). Parathyroid adenoma on the ipsilateral side of thyroid hemiagenesis. Proc (Bayl Univ Med Cent).

[8] Rosen RD, Bordoni B (2022). Embryology, parathyroid. StatPearls [Internet].

[9] Farndon JR (2001). Postoperative complications of parathyroidectomy. Surgical Treatment: Evidence-Based and Problem-Oriented.

[10] De Gregorio L, Lubitz CC, Hodin RA, Gaz RD, Parangi S, Phitayakorn R, Stephen AE (2016). The truth about double adenomas: incidence, localization, and intraoperative parathyroid hormone. J Am Coll Surg.

